# Increased Expression of Neprilysin Is Associated with Inflammation in Preeclampsia

**DOI:** 10.1007/s43032-023-01410-w

**Published:** 2023-12-19

**Authors:** Sara Atta, Rehab Mekky, Mostafa Ibrahim, Mohamed M. Abdallah, Mona A. H. Elbaz, Eman Radwan

**Affiliations:** 1 Medical Biochemistry Department, Faculty of Medicine, Assiut University, Assiut, Egypt; 2 Institute for Drug Development and Innovation Research, Assiut University, Assiut, Egypt; 3 Department of Obstetrics and Gynecology, Assiut University, Assiut, Egypt; 4 Biochemistry Department, Sphinx University, New Assiut, Assiut, Egypt

**Keywords:** Neprilysin, Preeclampsia, Inflammatory mediators

## Abstract

Preeclampsia (PE) is associated with a finely tuned equilibrium between trophoblast cell invasion and fetal-maternal immunological tolerance. An imbalance between proinflammatory (IL-6) and anti-inflammatory (IL-10) cytokines is a hallmark of PE. Neprilysin (NEP), a membrane-bound metalloprotease, is vulnerable to the inflammatory environment and plays a significant role in modulating vascular tone. The aim of this study was to determine the correlation between NEP (mRNA and protein) levels and the inflammatory status in PE patients compared to healthy pregnant women and to identify the role of NEP in evaluating the severity of preeclampsia. The study group comprised 52 pregnant women with PE while the control group comprised 47 normotensive pregnant women. After a caesarean section, placental tissue samples from patients and controls were collected to measure the expression levels of IL-6, TGF-β, IL-10, and NEP mRNA. In addition, an enzyme-linked immunosorbent assay was used to assess the quantity of NEP protein in blood samples. Our results revealed a significant positive correlation between NEP (mRNA and protein) and proinflammatory markers IL-6 and TGF-β levels in patients compared to controls and a significant inverse correlation between NEP and anti-inflammatory cytokine IL-10. Moreover, this is the first study to find a strong positive correlation between NEP level and PE severity. In conclusion, in PE patients, there is a substantial relationship between NEP, the degree of inflammation, and PE severity. NEP could act as a potential biomarker for diagnosis and prognosis of PE.

## Introduction

Preeclampsia (PE) is a pregnancy-associated hypertensive disorder and a major cause of perinatal morbidity and mortality. It is defined as new‐onset hypertension (systolic blood pressure ≥ 140 mmHg or diastolic blood pressure ≥ 90 mmHg) after 20 weeks of pregnancy accompanied by proteinuria (> 300 mg/24 h) or signs of maternal organ dysfunction [1]. PE is considered a prevalent disease linked to pregnancy with a reported frequency of 2 to 8% among pregnancies [2]. Furthermore, higher incidence rates are reported due to geographic, social, economic, and ethnic differences [2].

PE is characterized by immune imbalance that promotes an inflammatory state [3]. The immune equilibrium is mainly regulated by the signaling of interleukins 6 (IL-6) and 10 (IL-10), with a crucial role in the activation, proliferation, and differentiation of various types of inflammatory cells [3]. Transforming growth factor-β (TGF-β) is another cytokine with a role in controlling fetal-maternal immunological tolerance. TGF-β is essential for maintaining the immunosuppressive activity of T regulatory cells which has been linked to the PE etiology [3].

The precise orchestration of proinflammatory and anti‐inflammatory cytokines as well as the interaction between trophoblasts and immune cells is thought to be necessary for the formation and maintenance of a normal pregnancy. However, the cause of the elevated inflammatory response associated with PE requires further research.

Neprilysin (NEP) is a membrane-bound metalloprotease widely expressed on several cell types [4]. NEP has several peptide substrates found in the heart, blood vessels, nerve cells, and gastrointestinal and respiratory tracts [5, 6]. It binds and cleaves these peptides which include vasodilators as bradykinin and natriuretic peptides A, B, and C, consequently regulating the vascular tone [7]. NEP also has physiologic impacts on insulin regulation, beta-amyloid decomposition in the brain, and inflammation control [8].

Alterations in the placental expression of NEP were reported in PE which may affect the balance of various regulator peptides at the fetal-maternal interface [7]. The placenta also releases greater levels of NEP into the maternal circulation in PE. As NEP levels rise, hypertension develops, which is a crucial component of the perinatal illness PE [7].

As pregnancy is a very strictly controlled procedure, we aim to study the role of NEP in controlling the inflammatory pool and immune tolerance in abnormal pregnancies and to study its association with severity of PE.

## Subjects and Methods

### Patients’ Data

This study is a case control study conducted at the Gynecology and Obstetrics Department, Assiut Women’s Health Hospital, Egypt, and the Medical Biochemistry Department, Faculty of Medicine, Assiut University, Egypt, in the period between September 2022 and April 2023. An informed consent was obtained from each patient, and all the study procedures were approved by the Medical Ethics Committee (Institutional Review Board), Faculty of Medicine, Assiut University (approval number: 17101854). The patient group included 52 pregnant women, aged 20–40 years with gestational age > 28 weeks diagnosed with PE by blood pressure (≥ 140/90 mmHg) with proteinuria (≥ 300 mg/dL). The control group included 47 pregnant age-matched, normotensive women, with no proteinuria or other pregnancy disorders or complications and all of them gave birth to healthy infants. Hemoglobin (Hb) concentration in addition to liver, kidney, and platelet function was done for all study population. We excluded patients with gestational diabetes, pregestational hypertension (essential hypertension) or proteinuria, vaginal or intrauterine infection, major known fetal or chromosomal anomalies, multiple gestations, systemic illness, sepsis, or fever.

### Sample Collection and Handling

Placental tissue samples were surgically obtained from pregnant women under sterile conditions after caesarean section. Tissue samples (4 × 6 cm) were removed from the central region of the placenta and rinsed with cold phosphate buffered saline (PBS) and then quickly frozen and maintained at − 80 °C until required for real-time quantitative polymerase chain reaction (qPCR) analysis. Blood samples (2 mL) were collected in a plain test tube and centrifuged at 3000 rpm for 10 min, and then, serum was separated and stored at − 80 °C until time of enzyme-linked immunosorbent assay (ELISA) analysis.

### RNA Extraction and Real-Time qPCR

Total RNA was extracted from frozen tissue by using Gene JET RNA Purification Kit (catalog no. #K0731, Thermo Scientific Inc., USA) according to the manufacturer’s instructions. After quantitation using NanoDrop spectrophotometer, RNA (500 ng) was used for reverse transcription to complementary DNA (cDNA) with the High-Capacity cDNA Reverse Transcription Kit (catalog no. #K1622, Thermo Scientific, USA). cDNA was then amplified with Maxima SYBR Green qPCR Master Mix Kit (catalog no. #K0251, Thermo Scientific, USA) and used as a template for the genes in Table 1. A two-step reaction protocol was performed: an initial denaturation cycle of 95 °C for 10 min, followed by 40 amplification cycles of 95 °C for 15 s and 60 °C for 1 min using the Applied Biosystems 7500 Fast Real-Time PCR Machine (Applied Biosystems, Germany). Calculations were performed using the 2^−ΔΔCT^ method with GAPDH as reference gene (Table 1).

**Table 1:** Primer sequences

Gene	5′-3′ primer sequence
NEP	Forward: CTG CTG AGG GGT CAC GAT T Reversed: GAG TGC GAT CAT TTC ACA GC
IL-6	Forward: AAA GAG GCA CTG GCA GAA AA Reversed: TTT CAC CAG GCA AGT CTC CT
IL-10	Forward: TTG CAA AAC CAA ACC ACA AGA CA Reversed: TCT CGA AGC ATG TTA GGC AGG
TGF-β	Forward: TCC TGG CGA TAC CTC AGC AA Reversed: CTC AAT TTC CCC TCC ACG GC
GAPDH	Forward: ACA GTC AGC CGC ATC TTC TT Reversed: GAC AAG CTT CCC GTT CTC AG

### Enzyme-Linked Immunosorbent Assay (ELISA)

Neprilysin concentration was determined in serum samples using the Human NEP (Neprilysin) ELISA Kit (catalog no. E-EL-H0801, Elabscience, USA) according to the manufacturer’s instructions.

### Statistical Analysis

The statistical analysis was performed using Prism GraphPad Software 5.03. Data were statistically described in terms of mean ± standard deviation (± SD), or median and range when not normally distributed, frequencies and percentages when appropriate. Statistical comparison of differences between each two groups was analyzed using the Student *t* test for normally distributed data and Mann Whitney *U* test for non-normally distributed data. The chi-square (*χ*^2^) test was used to compare categorical data. Correlation analyses were performed using Spearman’s coefficient *U* tests. *p* value of less than 0.05 was considered statistically significant.

The sample size was calculated using G power software version 3.1.3 using *t* test for comparison difference between two independent means [9]. A hypothesized effect size 0.7 (difference between NEP expression between cases and controls) [10], alpha error probability 0.05, power 0.95 (1 − beta error probability), and allocation ratio 1:1 were employed. The minimum required sample size was found to be 90 (45 participants in each group).

## Results

### Clinical Characteristics of PE Patients

The present study included 52 patients. Their mean age was 29.15 ± 5.46 years (range, 20–40 years). Systolic and diastolic blood pressure (SBP) was 159.81 ± 12.6 and 98.1 ± 7.93 mmHg, respectively. The demographic and clinical characteristics of PE patients regarding age, residence, BMI, BP, history, gravidity, and onset of PE are summarized in Table 2.

**Table 2:** Clinical characteristics of PE patients and control subjects

Variables	Patients (*n* = 52)	Controls (*n* = 47)	*p* value
Age in years			
Mean ± SD	29.15 ± 5.46	27.40 ± 4.01	*0.075
Gestational age (week)	34.11 ± 3.19	36.85 ± 2.20	* < 0.001
Residence			
▪ Urban	16 (30.8%)	19 (40.4%)	**0.316
▪ Rural	36 (69.2%)	28 (59.6%)	
Blood pressure (mmHg)			
▪ Systolic	159.81 ± 12.6	114.67 ± 8.1	* < 0.001
▪ Diastolic	98.1 ± 7.93	76.38 ± 7.91	* < 0.001
Anthropometric measures			
▪ BMI (kg/m^2^)	27.25 ± 3.95	21.58 ± 3.07	* < 0.001
Gravidity			
▪ Primigravida	16 (30.8%)	8 (17.0%)	**0.111
▪ Others	36 (69.2%)	39 (83.0%)	
Onset of PE (week)			
▪ Late	32 (61.5%)		
▪ Early	20 (38.5%)		
History of PE	25 (48.1%)		

Data were expressed as frequency and % or mean ± SD

*PE* preeclampsia, *BMI* body mass index

*Independent sample *t* test was used to compare mean between groups. Values in bold indicate significance

**Chi-square was used to compare proportion between groups.Values in bold indicate significance

### Evaluation of Neprilysin in PE Patients

The results showed significant upregulation in relative mRNA expression of NEP in placenta tissues from PE patients compared to placenta tissue from control subjects (Table 3). Consistently, the circulating protein levels of NEP were also significantly increased in sera samples from PE patients compared to controls (Table 3). Furthermore, PE severity was found to significantly alter NEP mRNA and protein levels, with severe cases exhibiting greater levels than mild cases, as seen in Table 4. On the other hand, no significant differences were detected in NEP levels between patients with early or late onset of PE (Table 4).

**Table 3:** Expression of Neprilysin (mRNA and protein levels) in PE patients and control subjects

Variables	NEP mRNA level (fold change)	NEP protein level (ng/mL)
Patients (*n* = 52)	1.48 (0.0–8.22)	3.33 (1.81–4.40)
Controls (*n* = 47)	0.83 (0.07–2.50)	1.81 (0.25–2.20)
*p* value*	**0.033**	** < 0.001**

Neprilysin mRNA levels were assessed by real-time qPCR analysis. GAPDH was utilized to normalize expression data. Neprilysin serum protein expression was assessed by ELISA

*Mann Whitney *U* test was used to compare median between groups.  Values in bold indicate significance

**Table 4:** Comparison of Neprilysin mRNA and protein levels according to severity and onset of PE

Variables	Neprilysin mRNA level (fold change)	Neprilysin protein level (ng/mL)
Severity		
Mild (*n* = 24)	0.46 (0.0–2.75)	2.55 (1.80–3.41)
Severe (*n* = 28)	2.43 (0.53–8.22)	3.72 (2.99–4.40)
*p* value*	** < 0.001**	** < 0.001**
Onset		
Late onset	1.80 (0.001–6.77)	(2.11–4.4)
Early onset	0.99 (0.0–8.22)	3.13 (1.8–4.1)
*p* value*	0.176	0.201

*Mann Whitney *U* test was used to compare median between groups. Values in bold indicate significance

### Evaluation of Inflammation in PE

To investigate the association of inflammation with PE, we measured the relative mRNA expression of the inflammatory cytokines, TGF-β, IL-6, and IL-10 in placenta tissues from controls and patients. The results revealed a significant upregulation of TGF-β and IL-6 (Table 5) in placenta tissues from patients compared to control tissues. On the contrary, IL-10 mRNA levels were significantly downregulated in placenta tissues from patients compared to control tissues (Table 5).

**Table 5:** Expression of inflammatory markers (mRNA) in PE patients and control subjects

Variables	TGF-β mRNA level (fold change)	IL-6 mRNA level (fold change)	IL-10 mRNA level (fold change)
Patients (*n* = 52)	3.63 (0.00–17.88)	2.22 (0.01–11.96)	0.79 (0.17–5.31)
Controls (*n* = 47)	1.12 (0.21–3.56)	1.11 (0.22–3.04)	1.38 (0.28–5.66)
*p* value*	** < 0.001**	** < 0.001**	**0.024**

mRNA levels of TGF-β, IL-6, and IL-10 were assessed by real-time qPCR analysis. GAPDH was utilized to normalize expression data

*Mann Whitney *U* test was used to compare median between groups. Values in bold indicate significance

### Correlation Analyses

In the present study, we examined the correlations between NEP mRNA and protein levels, inflammatory cytokine expression (IL-6, IL-10, and TGF-β), BMI, PE severity, and gravidity. As shown in Table 6, the results showed that placental NEP mRNA and circulatory NEP protein levels were significantly positively correlated. In addition, both NEP mRNA and protein levels were significantly positively correlated with the expression levels of IL-6 and TGF-β, while they were negatively correlated with IL-10. However, both NEP mRNA and protein levels were not correlated with either BMI or gravida (Table 7).

**Table 6:** Correlation between NEP (mRNA and protein levels) and inflammatory markers in PE patients

Other parameters	Neprilysin mRNA level (fold change)		Neprilysin protein level (ng/mL)	
	*r*	*p* value	*r*	*p* value
Neprilysin mRNA level (fold change)			0.527	< **0.001**
Neprilysin protein level (ng/mL)	0.527	< **0.001**		
IL-6 mRNA level (fold change)	0.609	< **0.001**	0.604	< **0.001**
IL-10 mRNA level (fold change)	− 0.639	<** 0.001**	− 0.692	< **0.001**
TGF-β mRNA level (fold change)	0.628	< **0.001**	0.679	**< 0.001**

*r (Spearman correction coefficient); *p* value significant if < 0.05. Values in bold indicate significance

**Table 7:** Correlation between Neprilysin (mRNA and protein levels), BMI, and gravidity in PE patients

Other parameters	Neprilysin mRNA level (fold change)		Neprilysin protein level (ng/mL)	
	*r*	*p* value	*r*	*p* value
BMI	0.01	0.597	0.031	0.829
Gravida	0.157	0.111	0.182	0.196

*r (Spearman correction coefficient); *p* value significant if < 0.05

## Discussion

Preeclampsia (PE) is linked to a delicate balance between trophoblast cell invasion and fetal-maternal immunological tolerance which depends on the exact coordination and action of many immune cells and cytokines [11]. Placental ischemia and endothelial dysfunction are connected primarily by inflammatory processes [1].

Neprilysin (NEP) is an integral membrane-bound proteolytic metallopeptidase with a wide range of substrates [12]. NEP plays various physiological roles in cardiovascular regulation, immune response, and cell proliferation [12]. NEP effects also include the suppression of inflammation, the breakdown of natriuretic peptide in the heart and blood vessels, and the regulation of insulin [8].

NEP is susceptible to the proinflammatory environment [13]. Previous studies showed an association between elevated circulating levels of C-reactive peptide (CRP) and proinflammatory mediators such as interleukin-1 (IL-1) with raised matrix metalloproteases in different cell types [13]. Additionally, it was demonstrated that the placenta releases NEP into the maternal bloodstream at noticeably higher levels during PE, which can also increase the risk of hypertension and heart failure in the maternal system as well as the fetus [12]. Furthermore, it was recently reported that NEP inhibitors can attenuate oxidative stress, inflammation, and apoptosis [14]. Hence, in this study, we aimed to investigate the role of NEP as a matrix metalloprotease in controlling fetal-maternal immunological tolerance in abnormal pregnancies and to study its association with the severity of PE.

Our study results revealed a state of imbalance between proinflammatory and anti-inflammatory cytokines, with proinflammatory cytokines such as IL-6 being considerably higher in placental tissues of cases compared to controls and anti-inflammatory cytokines such as IL-10 being significantly lower in cases compared to controls. Moreover, in accordance with earlier studies [6, 15], we showed a positive correlation between proinflammatory indicators and NEP (mRNA and protein) levels in patients with PE.

Interestingly, our findings also showed that there is a substantial correlation between the severity of PE and both NEP placental mRNA levels and serum protein levels, which is relevant to the role of NEP in prognosis of PE. NEP could therefore be a biomarker for assessing severity.

The results of our research revealed increased level of TGF-β placental tissues of cases compared to controls that is also positively correlated to NEP levels. These results are in agreement with Zhang et al. who demonstrated that PE patients had higher levels of TGF-β in their decidua when compared to healthy controls [13, 16, 17]. Moreover, TGF-β levels and blood pressure have a positive correlation, according to a prior study by Ozkan et al. [18]. Another group of researchers examined the levels of serum TGF-β1 and the polymorphisms in the TGF-β1 gene promoter region and showed that women with PE had considerably higher serum TGF-β1 levels; however, there was no difference in TGF-β1 gene polymorphism between PE and control groups [19].

The exact cause of the elevated inflammatory response remains unclear; however, as reported previously, higher placental interleukin levels encourage excessive macrophage activation and inhibit the recruitment of decidual natural killer cells, which are essential for remodeling spiral arteries [20]. Furthermore, weakening of the decidual extracellular matrix through increased activity of matrix metalloproteinases, a key factor contributing to the development of PE, leads to further inhibition of trophoblast invasion [21]. This could account for the upregulation of NEP observed in our research participants as well as the increased expression of other inflammatory markers.

Inhibition of NEP has recently gained interest due to its role in the metabolism of natriuretic peptides and proinflammatory peptides [23, 24]. NEP inhibition is aimed at increasing natriuretic peptide levels and maintaining vasodilatation [25]. NEP inhibitors have shown therapeutic benefit in the treatment of a range of ailments, including cardiovascular and renal diseases [23, 24]. The combined use of NEP inhibitors and angiotensin-converting enzyme inhibitors has been effectively tested in the treatment of heart failure in non-pregnant people [26].

Investigating the effects of inhibition of NEP expression in PE patients presents a promising and potential therapeutic target for PE [10]. Therefore, we recommend further research to determine the efficacy of NEP inhibitors in the treatment and improvement of PE prognosis.

In conclusion, our results in line with previous research show that there is a positive correlation between NEP levels and the expression levels of IL-6 and TGF-β, while negatively correlating with IL-10. This emphasizes the association of NEP with the inflammatory status in PE patients.

## Strengths and Limitations

To our knowledge, the present study showed for the first time that there is a substantial correlation between both NEP placental mRNA levels and serum protein levels and the severity of PE. However, the relatively small sample size and lack of follow-up after delivery represent the main limitations of this study so future research with larger sample sizes and follow-up cases should be performed.

## Data Availability

All related data and materials are available from the corresponding author upon request.
